# Enoxaparin, Tinzaparin, and Apixaban Modulate Cancer Cell Procoagulant Activity and Viability: Comparison with Quercetin

**DOI:** 10.3390/cancers18111783

**Published:** 2026-05-29

**Authors:** Mohammed A. Baghdadi, Pedro Henrique Fernandes do Carmo Las Casas, Elisabeth Mbemba, Aurélie Rousseau, Prakasha Kempaiah, Andrey A. Svistunov, Victoria Bitsadze, Michèle Sabbah, Jawed Fareed, Patrick Van Dreden, Varvara Trachana, Eleftheria Lefkou, Grigoris Gerotziafas

**Affiliations:** 1 Research Group “Cancer–Angiogenesis–Thrombosis and Hemostasis”, Research Team “Cancer, Vessels, Biology and Therapeutics” (CaVITE), Saint-Antoine Research Center (CRSA), INSERM UMR_S_938, Saint-Antoine University Hospital, University Institute of Cancerology (UIC), Assistance Publique–Hôpitaux de Paris (AP-HP), Sorbonne University, 75012 Paris, France; 2 Research Center, King Faisal Specialist Hospital and Research Center, Jeddah 23433, Saudi Arabia; 3 Clinical Research Department, Diagnostica Stago, 92230 Gennevilliers, France; 4 Thrombosis and Hemostasis Research Laboratories, Center of Translational Research and Education (CTRE), Loyola University Stritch School of Medicine, Maywood, Chicago, IL 60153, USA; 5 Thrombosis Center, Department of Obstetrics, Gynecology and Perinatal Medicine, I.M. Sechenov First Moscow State Medical University (Sechenov University), 119991 Moscow, Russia; 6 Department of Biology, Faculty of Medicine, School of Health Sciences, University of Thessaly, 41110 Larissa, Greece; 7 Hematology-Transfusion Medicine Department, Faculty of Medicine, School of Health Sciences, University of Thessaly, 41110 Larissa, Greece; 8 VAS-European Independent Foundation in Angiology/Vascular Medicine, 20157 Milan, Italy

**Keywords:** low-molecular-weight heparin, quercetin, apixaban, thrombin generation, cancer-associated thrombosis, tissue factor

## Abstract

**Background/Objectives**: Tissue factor (TF)-expressing cancer cells and their extracellular vesicles (CaCe-dEVs) are key drivers of cancer-associated hypercoagulability and vascular dysfunction. While low-molecular-weight heparins (LMWHs) and direct FXa inhibitors are standard therapies for cancer-associated thrombosis, their direct effects on cancer cell procoagulant potential and endothelial responses remain incompletely defined. This study compared the impact of LMWHs (enoxaparin, tinzaparin), apixaban, and quercetin on cancer cell viability, thrombin generation, and CaCe-dEVs-induced endothelial injury. **Methods**: Pancreatic (BXPC3) and breast (MCF7) cancer cells and their vesicles were analyzed for TF expression and thrombin generation. Human umbilical vein endothelial cells (HUVECs) were pretreated with each agent prior to vesicle exposure. Cell viability, thrombin generation, and endothelial morphology were assessed using standard assays and microscopy. **Results**: Tinzaparin and quercetin significantly reduced cancer cell viability, whereas enoxaparin and apixaban showed no cytotoxicity. None of the agents affected HUVEC viability. All suppressed TF-mediated thrombin generation induced by cancer cells, with tinzaparin being most effective in BXPC3 cells. Quercetin exhibited a partial and limited protective effect on endothelial cells against CaCe-dEVs-induced dysfunction, while LMWHs and apixaban did not prevent endothelial damage. **Conclusions**: These findings suggest that LMWHs, apixaban, and quercetin modulate cancer-cell-driven hypercoagulability beyond anticoagulation, with quercetin and tinzaparin showing additional cytotoxic potential. Such dual effects may reduce thrombosis risk while impacting tumor progression, meriting further investigation.

## Simple Summary

Cancer patients are at increased risk of developing blood clots, a complication that contributes significantly to morbidity and mortality. This risk is associated with cancer cells that express tissue factors and release extracellular vesicles capable of activating blood coagulation and damaging the endothelial lining of blood vessels. Anticoagulant drugs such as low-molecular-weight heparins and direct factor Xa inhibitors are widely used to treat cancer-associated thrombosis, but their direct effects on tumor-related procoagulant mechanisms remain incompletely understood. In this study, we evaluated the effects of enoxaparin, tinzaparin, apixaban, and the quercetin on cancer cell procoagulant activity and endothelial responses. Our results show that tinzaparin and quercetin reduced cancer cell viability, while all agents decreased tissue-factor-dependent thrombin generation. Notably, quercetin showed a partial and limited protective effect on endothelial cells exposed to cancer-derived extracellular vesicles, displaying a mixed cellular phenotype. These findings suggest that certain anticoagulant agents and natural compounds may modulate tumor-driven hypercoagulability beyond their conventional anticoagulant action.

## 1. Introduction

Hypercoagulability occurs frequently in cancer patients and is associated with tumor aggressiveness, resistance to therapy, and cancer-associated thrombosis [1,2,3]. Cancer cells expressing tissue factor (TF) trigger thrombin generation, leading to fibrin network formation, creating protective clot “shields” that contribute to treatment resistance [4]. Newly diagnosed, treatment-naïve patients with cancer often show elevated markers of endothelial activation (e.g., VCAM-1, VWF, circulating endothelial cells), highlighting endothelial dysfunction as an early driver of tumor vascular remodeling [5,6,7].

Vascular remodeling promotes direct contact of cancer and endothelial cells, inducing “education” of the latter to support tumor growth, neo-angiogenesis, and metastasis. Tumor-neighboring endothelial cells display plasticity and may undergo endothelial-to-mesenchymal transition [8]. Cancer-cell-derived extracellular vesicles (CaCe-dEVs) are key mediators of this process, transferring oncogenic molecules such as microRNAs, proteins, and metabolites [9]. Endothelial cells exposed to CaCe-dEVs acquire a procoagulant phenotype [10].

Low-molecular-weight heparins (LMWHs) and oral direct inhibitors of activated factor X (FXa) (apixaban, edoxaban, rivaroxaban) are cornerstone agents in the prevention and treatment of cancer-associated thrombosis [11]. Multiple lines of evidence from modeling and translational studies indicate that LMWHs, beyond inhibiting thrombin generation, can interfere with cancer cell biology and exert anticancer activity [12,13,14,15,16], including inhibition of angiogenesis, tumor progression, and cell cycle regulation [13,14,17]. Direct FXa inhibitors have also been reported to display anticancer effects, with supratherapeutic concentrations of apixaban reducing proliferation and promoting apoptosis in experimental settings [18,19,20]. Beyond anticoagulants, quercetin is a dietary flavonoid that exhibits anti-inflammatory, antioxidant, and pro-apoptotic effects, and protects endothelial cells from hypoxia–reoxygenation injury [21,22]. Recent multi-omics and experimental evidence have further confirmed quercetin’s direct antitumor activity, demonstrating its capacity to inhibit cancer cell proliferation and migration through stable binding to key cell cycle regulatory targets [23]. Our previous studies showed that BXPC3 pancreatic and MCF7 breast cancer cells, and their CaCe-dEVs, express TF and induce thrombin generation, with BXPC3 demonstrating higher procoagulant activity [10,24,25]. Targeting TF and reversing endothelial cell “education” by CaCe-dEVs may therefore offer novel therapeutic strategies.

Building on this background, the present study aimed to determine whether LMWHs and direct oral anticoagulants (DOACs) have the following activities: (a) exert direct effects on cancer cells, beyond the inhibition of thrombin generation, by modulating cancer cell procoagulant activity and cell viability; (b) protect endothelial cells from the deleterious effects of exposure to CaCe-dEVs. To benchmark these effects, we performed a comparative analysis with quercetin, used here as a reference agent.

## 2. Materials and Methods

### 2.1. Cell Culture

Human pancreatic adenocarcinoma cells (BXPC3) and estrogen-receptor-positive breast adenocarcinoma cells (MCF7) were obtained from the American Type Culture Collection (ATCC, Rockville, MD, USA). These cell lines, differing in biological behavior and procoagulant potential, were selected to model high (BXPC3) and low (MCF7) procoagulant activity. These cell lines were selected based on their contrasting procoagulant profiles, with BXPC3 representing high and MCF7 representing low TF expression and procoagulant activity [25].

Using a previously published and validated experimental model [10], cells were cultured in a 96-well plate in RPMI-1640 media (Sigma-Aldrich (Merck), Guyancourt, France) at 50 cells/μL (100 μL/well) and incubated at 37 °C in a humidified 5% CO_2_ atmosphere until ~80% confluence before experimentation. Primary human umbilical vein endothelial cells (HUVECs) were purchased from Lonza (Levallois-Perret, France) and cultured in endothelial basal medium-2 (EBM-2, Clonetics) supplemented with 2% fetal bovine serum. Second-passage HUVECs were seeded in a 96-well plate at 50 cells/μL (100 μL/well) and incubated at 37 °C in a humidified 5% CO_2_ atmosphere until ~80% confluence before experimentation.

### 2.2. Cancer-Cell-Derived Extracellular Vesicle Isolation and Quantification

The CaCe-dEVs were isolated from conditioned media of confluent BXPC3 or MCF7 cultures via differential centrifugation to remove cells and debris as described previously [10]. The TF-dependent procoagulant activity of CaCe-dEVs in the culture supernatants was quantified using the ZYMUPHEN™ MP-Activity kit (Hyphen Biomed, Neuville-sur-Oise, France) according to a predefined protocol. Four centrifugation protocols were systematically compared, and the protocol yielding the highest EV yield with the lowest apoptotic body contamination was selected. EV preparations were analyzed by flow cytometry using calibration beads for size estimation and counting beads for absolute concentration. PS expression was confirmed by Annexin V-PE labeling and ELISA, as described in Section 2.8. The isolation protocol followed the nomenclature and recommendations of ISEV/MISEV2018 [26]. The TF-dependent procoagulant activity of CaCe-dEVs was quantified using the ZYMUPHEN™ MP-Activity kit (Hyphen Biomed, Neuville-sur-Oise, France), according to a predefined protocol.

### 2.3. Endothelial Cell Exposure to CaCe-dEVs

In preliminary experiments, HUVEC (5 × 10^5^ cells per 25 cm^2^ flask) were incubated with increasing concentrations of EVs derived from BXPC3 or MCF7 cells (120, 240, and 360 nM) for 24, 48, or 72 h. In preliminary experiments, the CaCe-dEVs at concentrations of 120 or 240 nM, incubated for 24, 48, or 72 h, did not induce any detectable changes, morphological changes, or TF expression in endothelial cells [27]. In contrast, exposure of HUVECs to 360 nM of CaCe-dEVs for 72 h induced marked morphological changes in the endothelial cells. Consequently, for all subsequent experiments, HUVECs were incubated with CaCe-dEVs at 360 nM for up to 72 h, following the validated protocol [10]. HUVECs were exposed to 360 nM BXPC3- or MCF7-dEVs, resuspended in 1 mL EBM-2, and incubated in 25 cm^2^ flasks for 72 h. The concentration of 360 nM for 72 h was established as the minimum biologically effective concentration based on systematic dose-escalation experiments and was applied in all subsequent experiments.

### 2.4. Pretreatment of HUVEC with Antithrombotic Agents

HUVECs (5 × 10^5^ cells per 25 cm^2^ flask) were pretreated for 24 h with the following treatments: 1 or 2 anti-Xa IU/mL concentrations of the LMWHs, enoxaparin (Lovenox^®^; Sanofi-Aventis France, Sanofi Winthrop Industrie, Le Trait, France) or tinzaparin (Innohep^®^, Laboratoires LEO, Vernouillet, France); 1 or 2 μg/mL concentration of the direct specific FXa inhibitor apixaban (Eliquis^®^; Bristol-Myers Squibb/Pfizer EEIG, Dublin, Ireland); 166.5 or 333 μM of quercetin (Sigma-Aldrich, St. Louis, MO, USA; Cat. No. Q4951; purity ≥ 95% by HPLC); PBS (control). All were diluted in EBM-2 medium. After 24 h, the cells were washed three times with PBS; subsequently, 360 nM of CaCe-dEVs suspended in EBM-2 medium were added for incubation periods of up to 72 h. No residual anti-Xa activity from enoxaparin or tinzaparin, nor any measurable apixaban, was detected in the washing solution after the third wash of cells previously exposed to these antithrombotic agents.

### 2.5. Thrombin Generation Assay

Thrombin generation was assessed according to a previously published validated experimental procedure [4] using the calibrated automated thrombogram (CAT) with the Thrombinoscope system and Thrombinoscope™ software version 5.0.0.742 (Diagnostica Stago, Asnières-sur-Seine, France) as follows: BXPC3, MCF7, or HUVECs (pretreated or not by agents and/or exposed to CaCe-dEVs) were seeded in 96-well plates (100 μL at 50 cells/μL). After 24 h of incubation, the wells were washed three times with warm PBS; then, 80 μL of normal platelet-poor plasma (PPP), purchased from Diagnostica Stago (Asnières-sur-Seine, France), was added. Thrombin generation was initiated by adding 20 μL of a triggering solution containing CaCl_2_ and fluorogenic substrate according to the manufacturer’s instructions. The following thrombogram parameters were analyzed: lag time, peak of thrombin (Peak), and endogenous thrombin potential (ETP).

### 2.6. Viability and Proliferation Assays

HUVECs (5 × 10^3^ cells/well) were incubated with CaCe-dEVs for 72 h. Cellular metabolic activity was assessed using the 3-(4,5-Dimethylthiazol-2-yl)-2,5-diphenyltetrazolium bromide (MTT) assay: (Roche Diagnostics, Mannheim, Germany), and absorbance was measured at 570 nm with a reference wavelength of 750 nm. The assay specifically measures mitochondrial reductase activity in metabolically active cells and allows for the evaluation of subtle changes in endothelial cell function following CaCe-dEVs exposure.

Cancer cells (5 × 10^3^ cells/well) were treated with test agents and incubated up to 72 h before staining with crystal violet (Sigma-Aldrich, St. Louis, MO, USA). Absorbance was measured at 570 nm. This assay was used for cancer cell analysis to quantify total adherent cell biomass, capturing both proliferative capacity and treatment-induced detachment.

HUVECs were exposed to CaCe-dEVs for 72 h. Following exposure, cells were harvested, washed with cold PBS, and incubated with propidium iodide (PI) solution according to the manufacturer’s instructions. Samples were then immediately analyzed by flow cytometry (FACS), and the percentage of PI-positive cells was quantified as a measure of necrotic cell rate.

### 2.7. Tissue Factor Expression

The TF expression was analyzed by flow cytometry using the murine anti-human TF antibody conjugated to FITC (ref 4508CJ, Sekisui Diagnostics, London, UK) and by ELISA using the ZYMUPHEN™ MP-TF kits (Hyphen Biomed, Neuville-sur-Oise, France) according to the manufacturer’s instructions.

### 2.8. Phosphatidylserine Expression

Phosphatidylserine (PS) expression was evaluated by flow cytometry using Annexin V conjugated to phycoerythrin (Sigma-Aldrich, St. Louis, MO, USA) and by ELISA using ZYMUPHEN MP Activity™ kits (Hyphen Biomed, Neuville-sur-Oise, France) according to the manufacturer’s instructions.

### 2.9. Microscopy

Cell morphology was visualized using an IX83 Olympus inverted microscope with a LUCPLFLN 20× PH objective and an ORCA-Flash 4.0 LT camera (Hamamatsu Photonics, Hamamatsu, Japan). Images were acquired and analyzed using CellSens Dimension software (v1.16, Olympus, Hamburg, Germany).

Confluence was assessed on inverted microscopy images using automated image segmentation of adherent cancer cells. Cell-covered area was quantified and expressed as the percentage of the total imaged surface occupied by cells, according to the following formula: Confluence (%) = cell-covered area/total image area × 100.

### 2.10. Statistical Analysis

Data are presented as mean ± standard deviation (SD) from 6 independent experiments. Normality was confirmed, and comparisons were made using repeated-measures one-way ANOVA. A *p*-value < 0.05 was considered statistically significant. Analyses were conducted using SPSS (v21.0; IBM Corp., Armonk, NY, USA).

## 3. Results

### 3.1. CaCe-dEVs Induced Alterations in the Procoagulant State of Endothelial Cells

**Figure 1:**
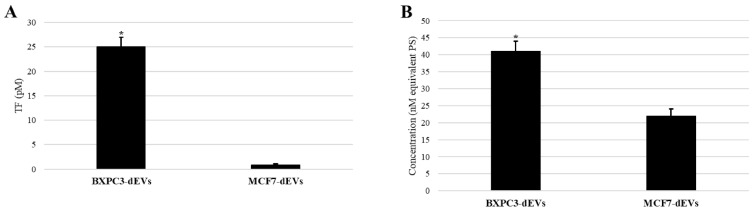
Expression of tissue factor (TF) (**A**) and procoagulant phospholipid (**B**) by HUVECs following exposure to CaCe-dEVs. Native HUVECs did not express detectable TF and expressed less than 5 nM PS. * *p* < 0.05 Comparing HUVECs exposed to BXPC3-dEVs versus HUVECs exposed to MCF7-dEVs.

Native HUVECs did not express any detectable levels of TF. Exposure of HUVECs to CaCe-dEVs induced significant TF expression. Upon treatment with BXPC3-dEVs, HUVECs exhibited markedly higher TF levels as compared to those treated with MCF7-dEVs (25 ± 2 pM vs. 0.9 ± 0.2 pM, respectively; *p* < 0.05) (Figure 1A).

Native HUVECs released less than 5 nM of EVs expressing phosphatidylserine (PS). Upon exposure to BXPC3- or MCF7-derived EVs, the concentration of PS-expressing EVs in the conditioned media increased to 41 ± 3 and 22 ± 2 nM, respectively, after 72 h (Figure 1B).

**Figure 2:**
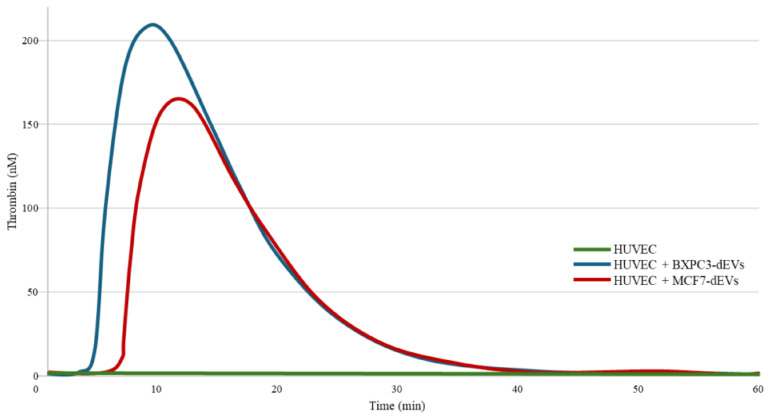
Representative thrombin-generation curves measured by calibrated automated thrombography (Thrombinoscope) in normal human platelet-poor plasma in contact with HUVECs following exposure to BXPC3-dEVs or MCF7-dEVs. Parameters derived from the curves include lag time, peak of thrombin, and endogenous thrombin potential. Data are representative of six independent experiments.

Native HUVECs did not induce any detectable thrombin generation in normal PPP. The HUVECs exposed to BXPC3-dEVs or MCF7-dEVs triggered thrombin generation. The thrombin generation lag time was significantly shorter in HUVECs exposed to BXPC3-dEVs compared with those exposed to MCF7-dEVs, whereas ETP and Peak did not differ significantly. Data are summarized in Figure 2, and Table 1 shows representative thrombin-generation curves induced by endothelial cells exposed to BXPC3-dEVs and MCF7-dEVs.

### 3.2. CaCe-dEVs Induced Alterations in the Functional State of Endothelial Cells

The confluence of HUVECs exposed to CaCe-dEVs for 72 h was significantly lower (20%) than that of native HUVECs (90%; *p* < 0.05). Native HUVECs exhibited significantly lower necrotic cell rates compared to those exposed to CaCe-dEVs, either BXPC3-dEVs or MCF7-dEVs (2.84% versus 7.21%, 5.12%, respectively; *p* < 0.05). Otherwise, no differences were observed in necrotic cell rates in HUVECs exposed to BXPC3-dEVs or MCF7-dEVs (*p* > 0.05).

**Figure 3:**
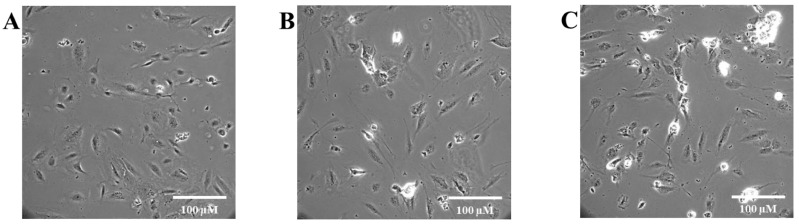
Morphological changes in HUVECs induced by exposure to CaCe-dEVs. (**A**) Native HUVECs; (**B**) HUVECs exposed to BXPC3-dEVs; (**C**) HUVECs exposed to MCF7-dEVs. Representative images from one of six independent experiments.

Phase-contrast microscopy showed clear morphological changes in the HUVECs after exposure to CaCe-dEVs compared with native cells. Morphological assessment was performed at 72 h, as this time point was selected to capture the progressive and sustained nature of CaCe-dEV-induced endothelial changes following the plateau of proliferation inhibition observed at 24 h. Figure 3 depicts representative images of the morphological alterations of HUVECs induced by exposure to CaCe-dEVs. Native HUVECs (Figure 3A) displayed the expected phenotype of quiescent endothelial cells with predominantly adherent, well-spread cells of elongated/spindle to polygonal morphology and a relatively homogeneous distribution across the field, with only rare rounded/refractile elements. Following exposure to BXPC3-dEVs (Figure 3B), HUVECs largely remained adherent and elongated, but they appeared less uniform, with increased morphological heterogeneity, more refractile/rounded cells, and occasional small aggregates, consistent with mild cellular stress or partial detachment in a subset of cells. Exposure to MCF7-dEVs (Figure 3C) produced the most marked alterations, characterized by a higher frequency of rounded, bright/refractile elements and clusters, with evidence of reduced spreading and focal disruption of the adherent monolayer, suggesting greater impairment of cell adhesion and viability compared with both native HUVECs and BXPC3-dEVs-treated cells.

Exposure of HUVECs to CaCe-dEVs significantly reduced cell proliferation. Preliminary experiments showed that, following incubation with 360 nM BXPC3-dEVs or MCF7-dEVs, the maximal reduction in HUVEC proliferation was reached after 24 h of exposure, indicating a plateau of the inhibitory effect. Although the maximal inhibitory effect was reached at 24 h, the experiment was extended to 72 h to assess whether this effect was sustained over time. Subtle but consistent inhibitory effects remained detectable throughout this period, which are biologically relevant in the context of sustained endothelial dysfunction induced by CaCe-dEVs. At 72 h, proliferation was reduced by 52 ± 5% and 37 ± 6% in the HUVECs exposed to BXPC3-dEVs and MCF7-dEVs, respectively (*p* < 0.05).

### 3.3. Modulation of Cancer-Cell-Driven Thrombin Generation by LMWHs, Apixaban, and Quercetin

In BXPC3 cells, enoxaparin (1 or 2 anti-Xa IU/mL) did not significantly modify lag time, ETP, or Peak compared with untreated controls. In MCF7 cells, enoxaparin at 1 anti-Xa IU/mL had no significant effect, whereas 2 anti-Xa IU/mL significantly reduced ETP (−24%) and Peak (−36%).

Tinzaparin at 1 anti-Xa IU/mL did not significantly affect thrombin generation in either cell line. At 2 anti-Xa IU/mL, tinzaparin significantly prolonged the lag time by 1.1-fold in both BXPC3 and MCF7 cells. In BXPC3 cells, this was accompanied by a 19% reduction in Peak, while the 8% decrease in ETP did not reach statistical significance. In MCF7 cells, 2 anti-Xa IU/mL tinzaparin significantly reduced ETP (−22%) and Peak (−32%).

Apixaban (1 or 2 μg/mL) did not significantly modify thrombin generation parameters in BXPC3 cells. In MCF7 cells, 1 μg/mL had no effect, whereas 2 μg/mL significantly prolonged the lag time (1.2-fold) and reduced ETP (−23%) and Peak (−30%).

Quercetin at 166.5 μM did not significantly affect thrombin generation in either cell line. At 333 μM, quercetin significantly prolonged the lag time in BXPC3 (1.2-fold) and MCF7 cells (1.6-fold). In BXPC3 cells, this was associated with a significant 11% reduction in Peak, while the 8% decrease in ETP was not statistically significant. In MCF7 cells, the higher concentration significantly reduced both ETP (−31%) and Peak (−41%) (Table 2).

### 3.4. Impact of LMWHs, Apixaban, and Quercetin on Cancer Cell Viability

Tinzaparin significantly reduced adherent cell biomass in both cell lines. At 1 anti-Xa IU/mL, adherent cell biomass decreased by 37% in BXPC3 cells and 18% in MCF7 cells. Increasing the concentration to 2 anti-Xa IU/mL further reduced adherent cell biomass by 46% and 29%, respectively (*p* < 0.05 vs. untreated controls).

In contrast, neither apixaban (2 μg/mL) nor enoxaparin (2 anti-Xa IU/mL) significantly affected cell adherent cell biomass.

**Figure 4:**
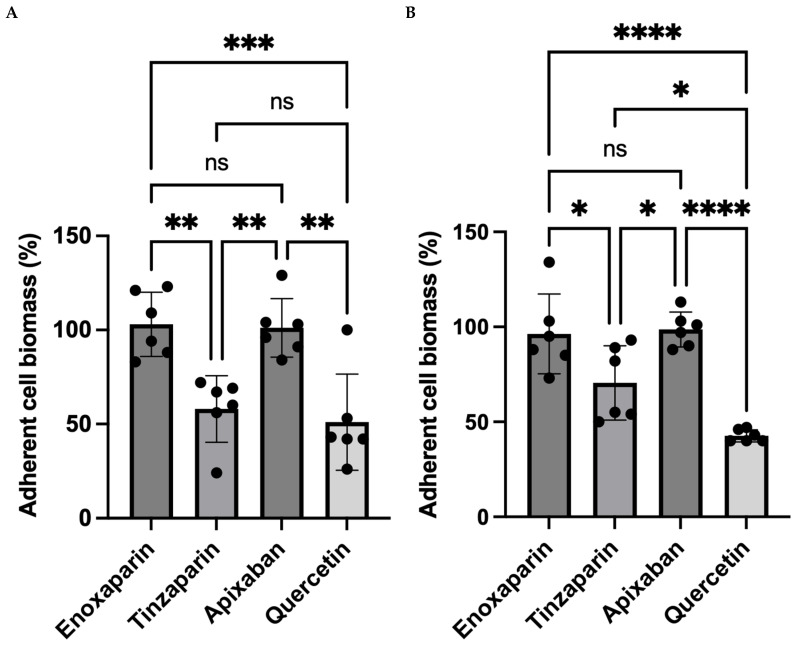
Adherent cell biomass after exposure to antithrombotic agents or quercetin. (**A**) BXPC3 and (**B**) MCF7 adherent cell biomass following 72 h incubation with enoxaparin or tinzaparin (2 anti-Xa IU/mL), apixaban (2 μg/mL), or quercetin (333 μM). Adherent cell biomass is expressed as a percentage of the untreated control. Data are presented as mean ± SD from six independent experiments. * *p* < 0.05 versus untreated control. ** *p* < 0.01, *** *p* < 0.001, and **** *p* < 0.0001 correspond to comparisons between different treatment conditions; ns = not significant.

Quercetin (333 μM) significantly reduced adherent cell biomass by 55% in BXPC3 cells and 39% in MCF7 cells (*p* < 0.05 vs. untreated cells) (Figure 4). Kinetic analyses demonstrated that the maximal effects of tinzaparin and quercetin on cancer-cell-adherent cell biomass were observed after 72 h of exposure.

### 3.5. Effects of Antithrombotic Agents and Quercetin on HUVEC Morphology and Protection Against CaCe-dEVs-Induced Alterations

**Figure 5:**
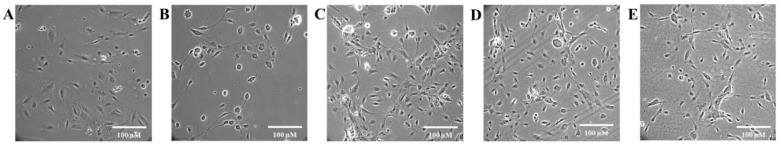
Morphological changes in HUVECs treated with enoxaparin or tinzaparin (2 anti-Xa IU/mL), apixaban (2 µg/mL), or quercetin (333 µM). (**A**) Native HUVECs, (**B**) HUVECs treated with enoxaparin, (**C**) HUVECs treated with tinzaparin, (**D**) HUVECs treated with apixaban, (**E**) HUVECs treated with quercetin. Representative images from one of six independent experiments.

Treatment of native HUVECs with enoxaparin, tinzaparin, apixaban, or quercetin at the tested concentrations did not significantly affect cellular metabolic activity compared with untreated controls. Morphological analysis revealed distinct patterns of endothelial remodeling. HUVECs treated with enoxaparin or tinzaparin displayed an elongated, spindle-shaped morphology with prominent cytoplasmic extensions and increased cell density relative to untreated cells (Figure 5A–C). Apixaban-treated HUVECs exhibited a comparable elongated phenotype, although cytoplasmic projections were less pronounced (Figure 5D). In contrast, quercetin-treated HUVEC appeared more rounded, with limited cytoplasmic extensions, closely resembling the morphology of untreated controls (Figure 5E).

**Figure 6:**
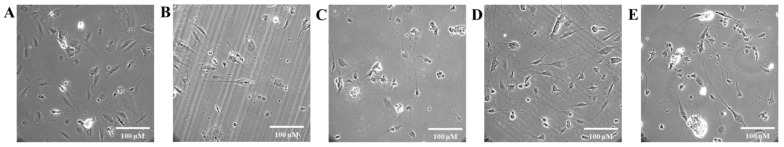
Morphological changes in HUVECs pretreated with enoxaparin or tinzaparin (2 anti-Xa IU/mL), apixaban (2 µg/mL), or quercetin (333 µM) and then exposed to BXPC3-dEVs. (**A**) Native HUVECs exposed to BXPC3-dEVs, (**B**) HUVECs pretreated with enoxaparin and exposed to BXPC3-dEVs, (**C**) HUVECs pretreated with tinzaparin and exposed to BXPC3-dEVs, (**D**) HUVECs pretreated with apixaban and exposed to BXPC3-dEVs, (**E**) HUVECs pretreated with quercetin and exposed to BXPC3-dEVs. Representative images from one of six independent experiments.

**Figure 7:**
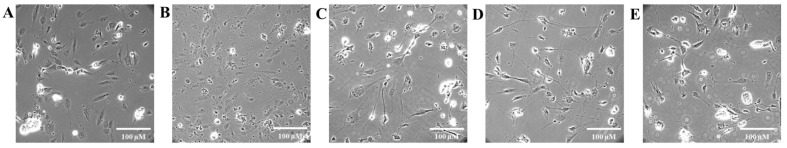
Morphological changes in HUVECs pretreated with enoxaparin or tinzaparin (2 anti-Xa IU/mL), apixaban (2 µg/mL), or quercetin (333 µM) and then exposed to MCF7-dEVs. (**A**) Native HUVECs exposed to MCF7-dEVs, (**B**) HUVECs pretreated with enoxaparin and exposed to MCF7-dEVs, (**C**) HUVECs pretreated with tinzaparin and exposed to MCF7-dEVs, (**D**) HUVECs pretreated with apixaban and exposed to MCF7-dEVs, (**E**) HUVECs pretreated with quercetin and exposed to MCF7-dEVs.

Pretreatment with enoxaparin or tinzaparin (1 or 2 anti-Xa IU/mL) did not significantly attenuate the morphological alterations induced by subsequent exposure to BXPC3-dEVs or MCF7-dEVs. As shown in Figure 6A–C and Figure 7A–C, respectively, the morphologies of pretreated HUVECs were comparable to those of cells exposed to BXPC3-dEVs or MCF7-dEVs alone.

In contrast, HUVECs pretreated with apixaban and then exposed to BXPC3-dEVs (Figure 6D) or MCF7-dEVs (Figure 7D) exhibited morphological alterations of lesser magnitude compared with cells treated with CaCe-dEVs alone, suggesting a partial protective effect.

HUVECs pretreated with quercetin and subsequently exposed to BXPC3-dEVs (Figure 6E) or MCF7-dEVs (Figure 7E) displayed a mixed phenotype, combining features of quercetin exposure and CaCe-dEVs-induced injury. These cells showed marked elongation, increased cytoplasmic protrusions, and disrupted cell–cell contacts. Although morphological changes were slightly attenuated compared with CaCe-dEVs-treated cells, the overall protective effect of quercetin appeared limited.

### 3.6. Effects of Enoxaparin, Tinzaparin, Apixaban, and Quercetin on HUVEC-Driven Thrombin Generation Following CaCe-dEVs Exposure

Exposure of native HUVECs to enoxaparin, tinzaparin, apixaban, or quercetin at the tested concentrations did not significantly modify thrombin generation compared with untreated controls.

Pretreatment of HUVECs with apixaban (2 μg/mL) prior to exposure to BXPC3-dEVs significantly reduced the Peak and ETP. The lag time showed only minor, non-significant changes.

Pretreatment with quercetin (333 nM) before exposure to BXPC3-dEVs resulted in a significant prolongation of lag time and a significant reduction in Peak and ETP compared with BXPC3-dEVs exposure alone.

In contrast, pretreatment of HUVECs with apixaban or quercetin prior to exposure to MCF7-dEVs did not significantly alter lag time, ETP, or Peak relative to MCF7-dEVs exposure alone (Table 3).

## 4. Discussion

This study focuses on the modulation of the procoagulant potential of cancer cells and the prevention of procoagulant transformation of endothelial cells upon exposure to TF-expressing CaCe-dEVs. It provides original evidence to formulate a mechanistic approach on the interactions of LMWHs with the biology of cancer cells and endothelial cells. The data presented herein show that the procoagulant potential of cancer cells is decreased upon their exposure to the LMWH tinzaparin but not to enoxaparin. The specific direct FXa inhibitor apixaban induced a decrease in the mild procoagulant potential of the breast cancer cells MCF7 but not of the highly aggressive BXPC3. Quercetin, a flavonoid with metabolic activity, also decreased the procoagulant potential of both BXPC3 and MCF7 cells. Pretreatment of endothelial cells with apixaban or quercetin also partially prevented morphological changes induced by CaCe-dEVs, underscoring their potential to modulate cancer-associated hypercoagulability beyond inhibition of thrombin generation.

The present study aimed to model the effects of antithrombotic agents and quercetin on the interaction between CaCe-dEVs and endothelial cells as part of the systemic vascular network. Accordingly, experiments were conducted in HUVECs, a well-established model of classical endothelial biology. Exposure of endothelial cells to CaCe-dEVs induced TF expression, likely originating from the endothelial cells themselves, as previously shown [10]. Contact with TF-bearing vesicles may further enrich endothelial TF. Exposure also triggered morphological changes, reduced proliferation, and increased mortality of endothelial cells. These morphological changes in endothelial cells were associated with enhanced TF expression and the amplification of thrombin generation. Compared with native endothelial cells, those adjacent to tumors exhibit structural and functional abnormalities [8,28,29], a phenotype reproduced in our study following CaCe-dEVs exposure. Surviving cells appeared elongated with extensive cytoplasmic extensions, suggesting enhanced motility and migration, resembling “cancer-associated endothelial cells” [8]. Tumor-associated endothelial cells are characterized by abnormal gene expression profiles, chromosomal instability, aneuploidy, and other cytogenetic abnormalities [30,31]. Emerging evidence suggests that membrane-level interactions with CaCe-dEVs may contribute to the induction of these alterations, although the underlying molecular mechanisms remain insufficiently understood and warrant further investigation.

Collectively, these findings identify CaCe-dEVs as key drivers of procoagulant transformation of endothelial cells and warrant translational research to define biomarkers and therapeutic targets for monitoring endothelial dysfunction and thrombotic risk in cancer patients. This phenomenon likely constitutes an amplification loop of hypercoagulability, activating endothelial cells both locally within the tumor microenvironment and distantly in the vascular network.

In the second part of the study, we examined the effects of LMWHs, apixaban, and quercetin on cancer cell procoagulant activity and the activation of endothelial cells by CaCe-dEVs. Quercetin was included as a non-anticoagulant control because of its concentration-dependent biological effects. Indeed, quercetin at low concentrations exerts antioxidant activity, whereas at higher concentrations it displays pro-oxidant and cytotoxic effects on cancer cells. [32].

Exposure of cancer cells to enoxaparin, tinzaparin, and apixaban significantly reduced their capacity to initiate and amplify thrombin generation. Quercetin produced a similar effect. However, results varied between the two cell lines. The panel of anticoagulants exhibited differential inhibitory effects. While MCF7 cell procoagulant activity was susceptible to all agents (both LMWHs, apixaban, and quercetin), BXPC3 cell activity was only inhibited by tinzaparin and quercetin.

Tinzaparin and quercetin also reduced the cancer-cell-adherent biomass, whereas enoxaparin and apixaban had no effect. The observed differential effect between tinzaparin and enoxaparin on the cancer-cell-adherent biomass is mechanistically consistent with well-established structural distinctions between these two LMWHs. Tinzaparin, characterized by a higher mean molecular weight and broader oligosaccharide chain distribution, interacts with a wider range of heparin-binding proteins implicated in tumor survival, including VEGF, FGF2, heparanase, and selectins [33,34]. In addition, tinzaparin potently inhibits cancer-cell-induced thrombin generation, attenuating downstream PAR-1 and ERK signaling pathways critical for cancer cell proliferation and survival [35]. In contrast, enoxaparin’s more uniform structure and narrower binding profile result is substantially weaker modulation of these tumor-promoting pathways, providing a mechanistic basis for the absence of cytotoxicity observed in our study [33]. These reductions were specific to cancer cells, as endothelial viability cellular metabolic activity remained unaffected. It should be noted that the crystal violet staining method used in this study measures the total adherent biomass and cannot discriminate between genuine cytotoxicity, cell detachment, and growth arrest. Confirmation using cell-death-specific assays such as Annexin V/PI co-staining, caspase activation, or LDH release is recognized as an important direction for future investigations.

The cytotoxic effects of tinzaparin and quercetin were more pronounced in pancreatic BXPC3 cells than in breast cancer MCF7 cells, suggesting that treatment sensitivity may depend on cancer cell heterogeneity, intracellular signaling pathways, metabolic status, and variability in the mechanisms of action of antithrombotic agents.

Tinzaparin and quercetin reduced cancer cell viability under the in vitro conditions used in the present study. However, these findings should not be interpreted as evidence of established anticancer activity in a clinical or in vivo setting. Although in vitro viability assays provide valuable mechanistic information, they cannot reproduce the complexity of the tumor microenvironment, immune and stromal interactions, systemic pharmacokinetics, or drug bioavailability observed in vivo. Consequently, the present findings should be considered preliminary and hypothesis-generating rather than demonstrative of a direct antitumor effect. We therefore acknowledge this limitation of the study and emphasize the need for dedicated in vivo investigations to determine whether these biological effects translate into clinically meaningful anticancer activity. The variability in cytotoxic response observed between the two cancer cell lines may also be partially explained by the dual pharmacological profile of quercetin.

Quercetin does not function solely as an antioxidant; its activity is concentration-dependent [36,37]. At low concentrations, it acts as a reactive oxygen species (ROS) scavenger, reducing intracellular oxidative stress. Conversely, at the higher concentrations used in the present study, quercetin is known to exert a pro-oxidant effect [36,37]. This shift leads to a sharp increase in intracellular ROS, which can induce mitochondrial dysfunction and ultimately apoptosis—a mechanism frequently implicated in its anticancer properties [32,38]. We hypothesize that the concentration of quercetin utilized here exploits the already elevated basal ROS levels in cancer cells, pushing them over a critical threshold and triggering cell death via this pro-oxidant pathway.

The differential effects observed between HUVECs and cancer cells are particularly noteworthy. Although a similar pro-oxidant mechanism may also occur in endothelial cells, their non-transformed phenotype is associated with more efficient antioxidant defense systems and greater redox homeostasis, potentially rendering them less susceptible to oxidative-stress-induced cytotoxicity. [39]. Upon exposure to quercetin, HUVEC cells may activate compensatory pathways, such as the NRF2-mediated stress response, to manage the oxidative load, reduce inflammation, and promote survival, possibly after a transient cell cycle arrest [40]. In cancer cells, one would anticipate a distinct sub-G1 population, indicative of apoptosis. In contrast, HUVEC cells could show a temporary arrest, likely in the G1 phase, followed by recovery. The observed morphological changes in HUVEC cells support this concept. This is further supported by recent evidence demonstrating that cell cycle dysregulation, particularly through the CDK6/E2F1 axis, plays a pivotal role in cancer cell proliferation and survival, highlighting cell cycle arrest as a relevant anticancer mechanism [17]. However, this hypothesis warrants further investigation through detailed cell cycle analysis. It should be acknowledged that the in vivo relevance of quercetin’s effects at the concentrations used in this study would require targeted delivery strategies to achieve comparable tissue exposures. Further studies are warranted to better characterize the pharmacological profile of quercetin and to optimize its delivery in order to maximize its potential anticancer efficacy in a clinically relevant context.

Based on this ensemble of our findings, we conclude that:

(a) LMWH-induced cytotoxicity on cancer cells does not represent a uniform class effect but rather reflects the specific properties of individual agents.
(b) Cancer cell aggressiveness appears to influence treatment sensitivity, as BXPC3 cells exhibited greater susceptibility than MCF7 cells.
(c) Effects on cell survival are distinct from effects on procoagulant potential, indicating a lack of direct correlation.
(d) Reduction in cancer cell procoagulant activity by tinzaparin was comparable to that of quercetin.
(e) Variability in efficacy reflects the molecular characteristics of each agent more than their anticoagulant activity.

We then sought to determine whether pretreatment of endothelial cells with these agents could prevent CaCe-dEVs-induced procoagulant transformation. Neither LMWHs nor apixaban fully prevented deleterious morphological or proliferative changes. Previous studies indicate that tinzaparin can reduce endothelial adhesion and angiogenesis indirectly [40,41]. Under our experimental conditions, the most notable effects were observed with apixaban and quercetin. Microscopy analysis revealed slight protective changes, although this requires confirmation using more sensitive imaging approaches. Apixaban partially mitigated the procoagulant shift induced by BXPC3-dEVs, but not by MCF7-dEVs. Quercetin produced a similar, yet stronger protective effect. However, none of the treatments fully prevented CaCe-dEVs-induced endothelial dysfunction at the tested concentrations. The experiments were conducted in cancer cell cultures without clotting factors or natural inhibitors. This design allowed us to identify direct cellular effects of LMWHs and apixaban on cancer cells and endothelial cells. The study exposed whole cancer cells (not isolated EVs) to the agents in the thrombin generation assay. Consequently, the relative contribution to thrombin generation from cell-surface TF versus EV-associated TF released into the well during the assay incubation period could not be distinguished.

The ensemble of these observations leads to the conclusion that in the studied LMWHs, the chemical composition of the low-affinity material (LAM) for the antithrombin might play a determinant role in the modulation of cancer cell biology. This interpretation is based on indirect experimental evidence rather than direct analytical characterization of the LAM fractions, as no purified fraction comparisons were performed in the current study. The fact that the observed biological effects appearing at supratherapeutic levels underlines also the potential dissociation between the anticoagulant-specific and the multitargeted effect of the LMWHs. Based on these data, future pharmacological studies on a potential anticancer effect of heparins should focus on the low-affinity material for the antithrombin. Moreover, the rationality for the concentration of this LAM should not follow that of the clinically relevant anti-Xa activity.

Future studies should explore broader metabolic and cytoprotective mechanisms through which the LAM of LMWHs modulate the procoagulant potential of cancer cells and potentially protect endothelial cells from the deleterious effects of CaCe-dEVs. The development of dedicated experimental systems is required to better assess the effects of these agents, particularly the LAM of LMWHs, on the interactions between cancer cells, CaCe-dEVs, and endothelial cells within the tumor vascular microenvironment. Further quantitative morphometric analysis is needed to better characterize the observed morphological changes, and quantitative functional assays (such as endothelial barrier integrity assessment by TEER measurement, FITC–dextran permeability, wound-healing assay, or quantitative TF/PS expression analysis) will be required to strengthen these conclusions.

Systematic mechanistic investigations integrating multi-omics approaches, network pharmacology, and molecular docking analyses—as recently reported for other pharmacological agents with potential anticancer effects [42]—could provide a more comprehensive understanding of the molecular pathways and targets underlying the biological effects observed in the present study. Such integrative approaches may also help overcome some of the inherent limitations associated with the current in vitro experimental design. In addition, direct analytical characterization and experimental validation using purified LAM fractions will be required to confirm the proposed mechanisms and should therefore be addressed in future investigations.

The present study provides novel insights into the modulation of the procoagulant phenotype and the viability of cancer cells by LMWHs, apixaban, and quercetin, as well as into their effects on the interactions between procoagulant cancer-cell-derived extracellular vesicles (CaCe-dEVs) and endothelial cells. The primary objective of the study was to investigate the functional and biological consequences of these interactions rather than to perform an in-depth structural and molecular characterization of isolated CaCe-dEVs. Consequently, the experimental design was not intended to provide comprehensive biophysical or biochemical characterization of EV populations; thus, it does not include detailed assessments of vesicle size distribution, concentration, subtype heterogeneity, or molecular phenotype. This constitutes a limitation of the present study. Nevertheless, EV preparations were evaluated using functional and flow-cytometry-based approaches, including Annexin V labeling and procoagulant activity assays, thereby confirming the phosphatidylserine-positive vesicular nature of the isolated CaCe-dEVs. In addition, EV isolation procedures were performed according to the nomenclature and methodological recommendations of the ISEV/MISEV2018 guidelines [26].

These findings provide the rationale for a dedicated follow-up study aimed at an in-depth characterization of CaCe-dEVs using state-of-the-art methodologies, including biophysical characterization by Nanoparticle Tracking Analysis (NTA) or Tunable Resistive Pulse Sensing (TRPS) for accurate determination of vesicle size distribution and concentration, detailed biochemical characterization of CaCe-EV cargo and membrane composition, phenotypic profiling using canonical EV markers (CD9, CD63, CD81), and evaluation of potential contaminants such as apoptotic bodies, protein aggregates, and lipoproteins. Such complementary analyses will strengthen the mechanistic interpretation of the present findings and further clarify the role of antithrombotic agents and CaCe-dEVs in cancer-associated hypercoagulability and endothelial dysfunction.

## 5. Conclusions

In conclusion, our study offers novel experimental data showing that enoxaparin, tinzaparin, apixaban, and quercetin modulate the procoagulant potential of cancer cells and influence endothelial cell transformation induced by CaCe-dEVs. Tinzaparin and quercetin reduced adherent cell biomass, suggesting a potential cytotoxic effect of tinzaparin on cancer cells under the specific in vitro conditions tested. Whether this represents true antineoplastic activity requires confirmation in in vivo experimental models. Apixaban and quercetin provided partial protection against endothelial morphological and functional changes induced by exposure to CaCe-dEVs. Table 4 summarized the ensemble of the biological effects of the LMWHs, apixaban, and quercetin on cancer cells and endothelial cells. Mechanistic and translational studies will be required to confirm these effects and evaluate their clinical applicability.

## Figures and Tables

**Table 1:** Thrombin generation parameters measured by calibrated automated thrombography (Thrombinoscope) in normal human platelet-poor plasma in contact with HUVECs following exposure to BXPC3-dEVs or MCF7-dEVs. Parameters derived from the curves were lag time, peak of thrombin, and endogenous thrombin potential (ETP). Results are presented as mean ± standard deviation from six independent experiments. Native HUVECs did not induce detectable thrombin generation within 40 min. * *p* < 0.05 versus HUVEC + BXPC3-dEVs.

Parameters	Native HUVEC	HUVEC + BXPC3-dEVs	HUVEC + MCF7-dEVs
Lag time (min)	>40	4.0 ± 0.2	6.0 ± 0.1 *
ETP (nM·min)	0	1603 ± 367	1476 ± 437
Peak (nM)	0	214 ± 95	167 ± 82

**Table 2:** Effect of pretreatment of (**a**) BXPC3 and (**b**) MCF7 cancer cells with enoxaparin, tinzaparin, apixaban or quercetin on their ability to trigger thrombin generation in normal human platelet-poor plasma, assessed by calibrated automated thrombography (Thrombinoscope^®^). Data are shown as mean ± standard deviation from six independent experiments. Low concentrations: enoxaparin and tinzaparin, 1 anti-Xa IU/mL; apixaban, 1 µg/mL; quercetin, 166.5 µM. High concentrations: enoxaparin and tinzaparin, 2 anti-Xa IU/mL; apixaban, 2 µg/mL; quercetin, 333 µM. * *p* < 0.05 versus control; $ *p* < 0.05 versus enoxaparin, tinzaparin, or apixaban.

Parameters	Control		Enoxaparin		Tinzaparin		Apixaban		Quercetin	
	Low	High	Low	High	Low	High	Low	High	Low	High
**(a)**										
Lag time (min)	1.93 ± 0.1	2.33 ± 0.2	1.71 ± 0.0	2.06 ± 0.2	1.99 ± 0.1	3.00 ± 0.5 *	1.82 ± 0.1	2.06 ± 0.1	2.27 ± 0.1	2.72 ± 0.1 *
ETP (nM·min)	1009 ± 4.7	1452 ± 50	969 ± 28.1	1426 ± 90	1003 ± 29.5	1336 ± 50	976 ± 20.1	1529 ± 100	945 ± 37.5	1340 ± 50
Peak (nM)	119 ± 1.5	161 ± 10	121 ± 1.71	169 ± 6	120 ± 2.9	131 ± 7 *	122 ± 1.8	177 ± 20	108 ± 4.9	144 ± 6 *
**(b)**										
Lag time (min)	11.49 ± 0.1	11.84 ± 0.5	10.46 ± 1.52	12.78 ± 1.1	10.9 ± 0.0	13.33 ± 0.5	11.90 ± 1.43	14.33 ± 1.2 *	12.16 ± 0.83	18.61 ± 1 *
ETP (nM·min)	652 ± 47.4	945 ± 0.5	674 ± 23.86	717 ± 0.5 *	687 ± 9.41	735 ± 0.5 *	672 ± 97.11	729 ± 7 *	789 ± 38.11	649 ± 0.5 *$
Peak (nM)	41 ± 5	73 ± 7	43.63 ± 6.64	47 ± 5 *	50.40 ± 0.48	50 ± 5 *	51.93 ± 16.63	51 ± 5 *	67.21 ± 1.03	43 ± 5 *$

**Table 3:** Impact of HUVECs pretreated with apixaban (2 µg/mL) or quercetin (333 µM) and exposed to BXPC3- or MCF7-dEVs on thrombin generation. Data are shown as mean ± standard deviation from six independent experiments. * *p* < 0.05 versus HUVECs exposed to BXPC3-dEVs. $ *p* < 0.05 versus HUVEC/BXPC3-dEVs. ++ *p* < 0.05 versus native HUVEC.

	Not Exposed to CaCe-dEVs			Exposed to BXPC3-dEVs			Exposed to MCF7-dEVs		
Parameters	Native HUVEC	HUVEC + Apixaban	HUVEC + Quercetin	HUVEC	HUVEC + Apixaban	HUVEC + Quercetin	HUVEC	HUVEC + Apixaban	HUVEC + Quercetin
Lag time (min)	11.5 ± 2.9	12 ± 1.2	13 ± 1.0	4.0 ± 0.2 ++	5.0 ± 0.9	6.0 ± 1.0 *++	6.0 ± 0.1 *++	6.0 ± 0.5	6.5 ± 1.0
ETP (nM·min)	910 ± 150	940 ± 45	1002 ± 69	1603 ± 367 ++	1250 ± 120	1011 ± 60 ++	1476 ± 437 ++	1360 ± 77	1250 ± 190
Peak (nM)	60.7 ± 15	71 ± 8	75 ± 10	214 ± 95 ++	169 ± 6 $	177 ± 5 $	167 ± 82 *++	165 ± 50	159 ± 22

**Table 4:** Summary of the key effects of CaCe-dEVs and modulation by antithrombotic agents on endothelial and cancer cells.

Parameter	Effect of CaCe-dEVs on Endothelial Cells (HUVECs)	Effect of Antithrombotic Agents on Cancer Cells (BXPC3 and MCF7)	Protective Effects of Antithrombotic Agents and Quercetin on HUVECs Exposed to CaCe-dEVs
Procoagulant phenotype	CaCe-dEVs induced a marked endothelial procoagulant shift, characterized by increased TF expression, release of phosphatidylserine-positive EndCe-dEVs, enhanced thrombin generation. BXPC3-dEVs exerted stronger procoagulant effects than MCF7-dEVs.	Tinzaparin and quercetin reduced thrombin generation induced by both BXPC3 and MCF7 cells. Enoxaparin and apixaban reduced thrombin generation induced only by MCF7 cells, with no significant effect in BXPC3 cells.	Pre-exposure to apixaban and quercetin partially prevented the procoagulant shift of endothelial cells induced by exposure to BXPC3-dEVs. No significant protection was observed against MCF7-dEV-induced procoagulant shift.
Cell viability/proliferation	CaCe-dEVs significantly reduced endothelial cell viability and proliferation BXPC3-dEVs exerted stronger effects than MCF7-dEVs.	Tinzaparin and quercetin significantly reduced cancer cell viability in both BXPC3 and MCF7 cells. Enoxaparin and apixaban showed no significant cytotoxic effects.	No protective effect on endothelial cell viability or proliferation.
Cell morphology	CaCe-dEVs disrupted endothelial monolayer integrity, reduced confluence, and increased rounded/refractile cells, consistent with endothelial injury. MCF7-dEVs. induced more important morphological alteration s of endothelial cells than BXPC3-dEVs.	No significant morphological changes were assessed in cancer cells following treatment.	Pre-exposure to enoxaparin or tinzaparin failed to prevent endothelial cell morphological alterations induced by exposure to CaCe-dEVs. Apixaban limited attenuation, whereas quercetin provided modest and heterogeneous protection.

Abbreviations: CaCe-dEVs, cancer cell-derived extracellular vesicles; HUVECs, human umbilical vein endothelial cells; TF, tissue factor; EVs, extracellular vesicles; EndCe-dEV: endothelial cell-derived extracellular vesicles.

## Data Availability

The data supporting the findings of this study are available upon reasonable request from the corresponding author.
